# Radiation dose reduction in pediatric great vessel stent computed tomography using iterative reconstruction: A phantom study

**DOI:** 10.1371/journal.pone.0175714

**Published:** 2017-04-14

**Authors:** Annemarie M. den Harder, Dominika Suchá, Pieter J. van Doormaal, Ricardo P. J. Budde, Pim A. de Jong, Arnold M. R. Schilham, Johannes M. P. J. Breur, Tim Leiner

**Affiliations:** 1 Department of Radiology, Utrecht University Medical Center, Utrecht, The Netherlands; 2 Department of Radiology, Erasmus Medical Center, Rotterdam, The Netherlands; 3 Department of Pediatric Cardiology, Utrecht University Medical Center, Utrecht, The Netherlands; Northwestern University Feinberg School of Medicine, UNITED STATES

## Abstract

**Background:**

To study dose reduction using iterative reconstruction (IR) for pediatric great vessel stent computed tomography (CT).

**Methods:**

Five different great vessel stents were separately placed in a gel-containing plastic holder within an anthropomorphic chest phantom. The stent lumen was filled with diluted contrast gel. CT acquisitions were performed at routine dose, 52% and 81% reduced dose and reconstructed with filtered back projection (FBP) and IR. Objective image quality in terms of noise, signal-to-noise ratio (SNR) and contrast-to-noise ratio (CNR) as well as subjective image quality were evaluated.

**Results:**

Noise, SNR and CNR were improved with IR at routine and 52% reduced dose, compared to FBP at routine dose. The lowest dose level resulted in decreased objective image quality with both FBP and IR. Subjective image quality was excellent at all dose levels.

**Conclusion:**

IR resulted in improved objective image quality at routine dose and 52% reduced dose, while objective image quality deteriorated at 81% reduced dose. Subjective image quality was not affected by dose reduction.

## Introduction

Coarctation of the aorta is a common congenital heart disease. The most frequent treatment for coarctation of the aorta is stent implantation [1]. After implantation, imaging follow-up is needed to detect complications like in-stent stenosis and aneurysm formation [2–4]. Guidelines propose to perform regular follow-up with CT angiography or magnetic resonance imaging at intervals of less than five years [4]. CT angiography is often the modality of first choice, because it is fast, widely available, non-invasive, associated with less metal artefacts compared to magnetic resonance imaging and this part of the aorta is difficult to visualize with ultrasound [5]. A large multi-institutional study showed that CT is used more than five times as often as MRI for follow-up after stent implantation [1]. However, concerns about the harmful effect of radiation have led to an increased focus on radiation dose reduction. Especially since aortic coarctation stents are predominantly implanted in children [1], who are more radiosensitive and have a longer expected lifetime to develop stochastic effects [6]. Furthermore, the regular CT follow-up in those patients can lead to a substantial cumulative dose. A substantial radiation dose reduction can be achieved by optimizing acquisition parameters and using iterative reconstruction (IR) [7]. A recent study showed that IR allows for a 25–41% radiation dose reduction in pediatric CT angiography [7], while studies in adults reported radiation dose reductions of up to 48% with IR for coronary CT [8]. Two phantom studies investigating the use of IR for prosthetic heart valve imaging reported a radiation dose reduction of 50–75% without a decrease in objective image quality [9,10]. In the current study we investigated the achievable radiation dose reduction with IR for CT angiography of aortic coarctation stents. To assess whether substantial radiation dose reduction for pediatric CT angiography is feasible, we performed an *in vitro* study. We evaluated the effect of dose reduction on objective and subjective image quality of commonly used stents to treat coarctation aortae.

## Materials and methods

### Phantom

**Fig 1:**
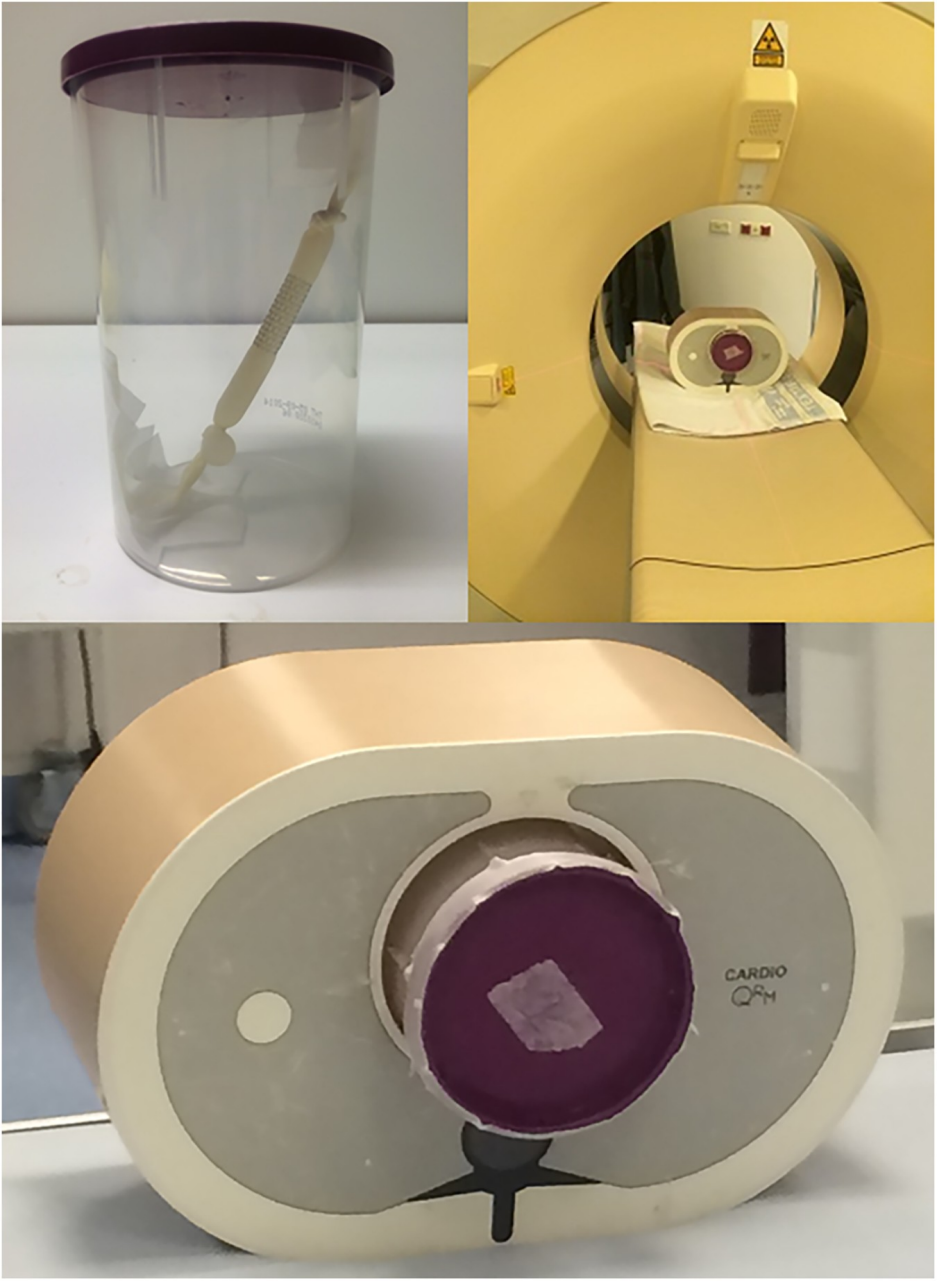
Phantom set-up. A plastic holder containing a stent with inside a balloon (left upper image) was placed into an anthropomorphic chest phantom (right upper image, bottom image) [2].

Five different stents were studied: (1) Advanta V12 covered stent made of stainless steel (Atrium Medica, length 25mm), (2) AndraStent 30-XL stent made of cobalt-chromium (Andramed, length 39mm), (3) Cheatham-Platinum stent made of 0.013” platinum / iridium wire (NuMED), (4) IntraStent Max LD made of stainless steel (EV3, length 36mm) and (5) Formula 535 stent made of 316L stainless steel (Cook Medical). An Atlas balloon (Bard BV) was used to inflate stent 1–4 to a diameter of 20 mm. Stent 5 (a premounted Formula 535 stent) was dilated to a diameter of 10 mm because this is the maximal vendor recommended size. To simulate a contrast-enhanced vessel, a balloon filled with diluted contrast-gel (20 times diluted) was placed inside every stent. The stents were placed at an angle of approximately 30 degrees in a plastic holder. The plastic holder was filled with gel without contrast and placed in a commercially available anthropomorphic chest phantom (D100, QRM GmbH, Moehrendorf, Germany) which simulates the radiation absorption of a small person [11]. The posterior-anterior distance is 200 mm and the distance from left to right is 300 mm. Images of the phantom setup are provided in Fig 1.

### CT acquisition protocol

A 256-slice CT scanner (Brilliance iCT, Philips Healthcare, Best, The Netherlands) was used for image acquisition. The following parameters were used: collimation 128 x 0.625 mm, slice thickness 0.9 mm, rotation time 0.27 seconds and a matrix size of 512 x 512 pixels. A standard sequential cardiac CT protocol was used for all protocols with an ECG generator to simulate a heart rate of 60 beats/min. The tube voltage was 100 kV for the routine dose protocol and 80 kV for the low dose protocols. The tube current-time product was 195 mAs for the routine dose protocol and 195 mAs and 80 mAs respectively for the low dose protocols. Each stent was scanned eight times per protocol with small translations of the phantom to take interscan variation into account, resulting in a total of 120 acquisitions. Images were reconstructed with filtered back projection (FBP) and IR (iDose^4^ level 3, Philips Healthcare, Best, The Netherlands). IDose^4^ has seven levels of noise reduction, with a higher level implicating more noise reduction. IDose^4^ level 3 was used as it is recommended by the vendor in this setting. Volumetric CT dose index (CTDI_vol_) based on a 32 cm phantom and dose-length product (DLP) were recorded for each scan. The scan length was 54 mm for the stents with a diameter of 20 mm and 73 mm for the stent with a diameter of 10 mm due to the length of this stent.

### Image quality

Objective image quality was assessed by drawing a homogeneous region of interest (ROI) in the gel surrounding the stent with a diameter of approximately 20 mm and a smaller ROI in the contrast within the stent. Average CT values (HU) and standard deviation (SD) were obtained. Based on this, noise, contrast-to-noise ratio (CNR) and signal-to-noise ratio (SNR) were calculated [12]. Noise was defined as the SD of the ROI and the SNR as the ratio between the mean HU and the SD. The SNR was calculated for the ROI in the contrast within the stent. The CNR was computed using the following equation:

$$CNR=\frac{HU(contrast\;within\;stent)-HU(gel)}{\sqrt{\frac{1}{2}x({SD(contrast\;within\;stent)}^{2}+{SD(gel)}^{2})}}$$

For each acquisition the relative difference in image quality was calculated as the percentage difference compared to FBP at routine dose (reference standard).

Subjective image quality was assessed at the center and at the outlets of the stent by two radiologists using a 4-point scale and standardized scoring forms:

1. Poor, non-diagnostic image quality, in stent lumen not delineated due to severe artifacts or excessive noise
2. Moderate, limited diagnostic value, stent lumen is assessable but partially obscured due to moderate artifacts or noise
3. Good, diagnostic image quality, stent skeleton and lumen delineated with minor artifacts or noise
4. Excellent, excellent image quality with clear delineation of stent and lumen without artifacts or noise.

Subjective image quality was evaluated by two observers using one acquisition of each CT protocol and observers were blinded for stent type and acquisition protocol. One observer was a radiologist with 6 years’ experience, the second observer was a pediatric cardiologist with 6 years’ experience in cardiovascular CT.

### Statistical analysis

SPSS Statistics version 20.0 for Windows was used for statistical analysis. Data were compared using the Friedman test and post-hoc analyses were performed with the Wilcoxon signed-rank test to test for significant differences compared with the reference standard namely the routine dose CT protocol reconstructed with FBP. A p-value <0.05 was considered statistically significant for the Friedman test and a Bonferroni correction was made for the post hoc Wilcoxon signed-rank test with a p-level set at 0.01. Inter-observer reproducibility for subjective image quality was assessed with the Cohen kappa coefficient and percentage of agreement. The kappa was interpreted as poor (k = 0.00–0.20), fair (k = 0.21–0.40), moderate (k = 0.41–0.60), good (k = 0.61–0.80) or excellent (k = 0.81–1.00). Statistic significant differences in subjective image quality were tested using the Wilcoxon signed-rank test with a p-level set at 0.05.

## Results

**Fig 2:**
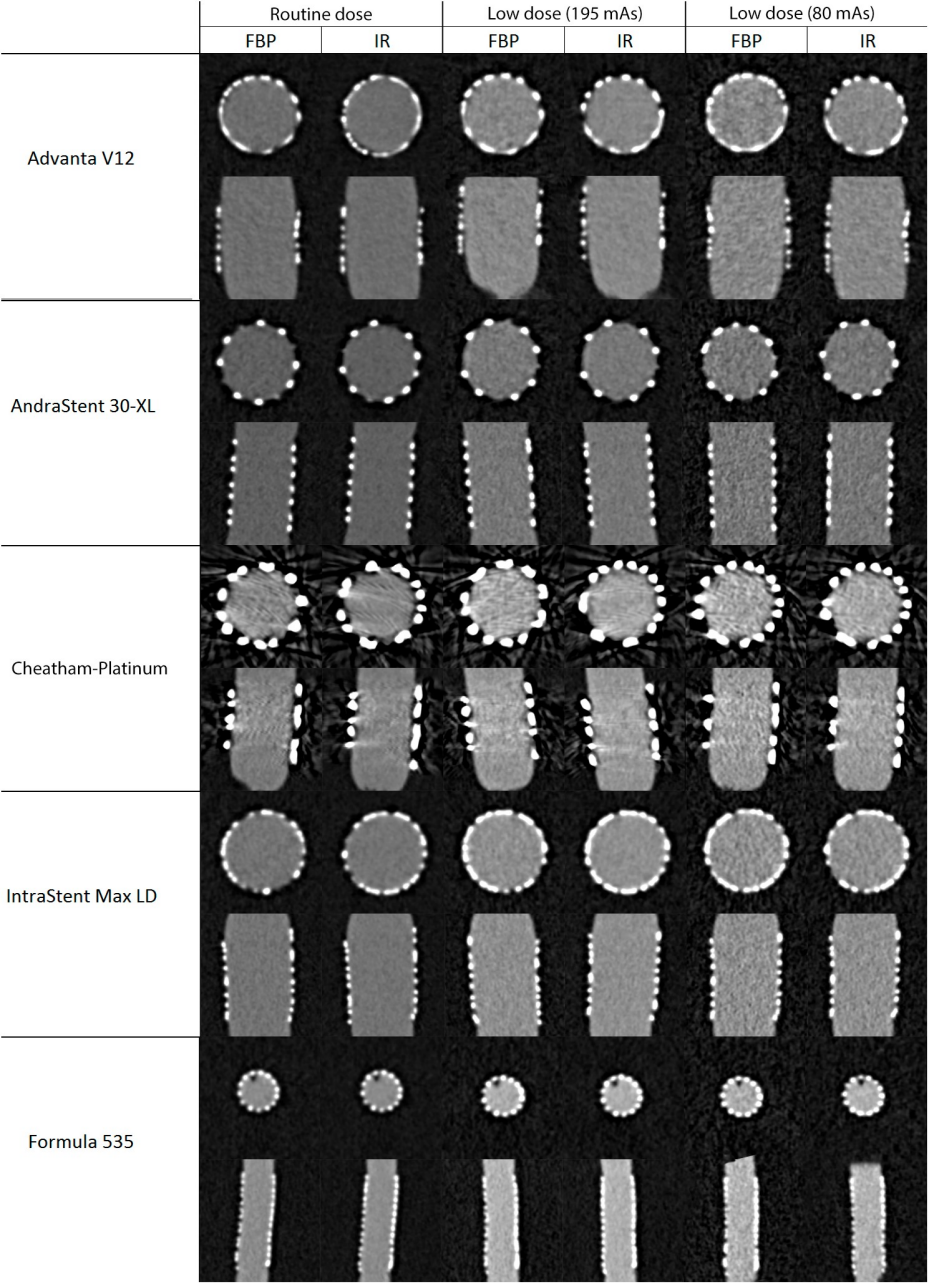
Example of the stents acquired with different protocols: routine dose (195 mAs, 100kV), low dose (195 mAs, 80kV), low dose (80mAs, 80kV).

An example of the stent images with different protocols is provided in Fig 2. The CTDIvol of the routine dose protocol (100 kV, 195 mAs) was 7.7 mGy while the low dose protocols were 3.7 mGy and 1.5 mGy respectively. This resulted in a relative dose reduction of 52% and 81% respectively. The DLP was 51.2 mGy*cm, 23.6 mGy*cm and 9.7 mGy*cm respectively. The average attenuation of the balloon, simulating the contrast-enhanced vessel, was 875 HU and the average attenuation of the gel surrounding the balloon was 10 HU.

### Objective image quality

**Fig 3:**
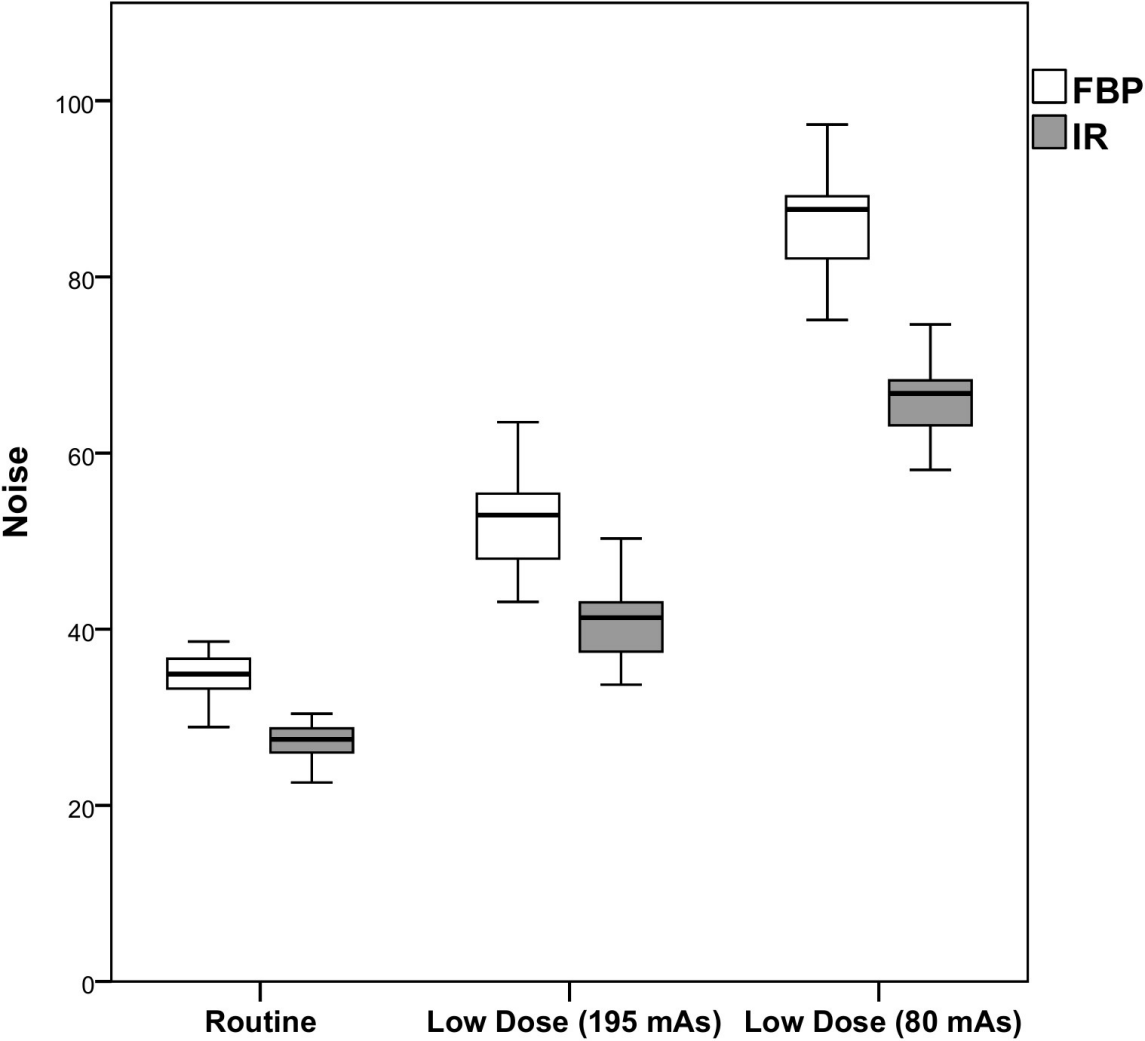
Noise per dose level for FBP and IR. The white boxes represent the noise with FBP, while the gray boxes represent the noise with IR. IR resulted in a decrease in noise compared to FBP at the same dose level. *FBP Filtered Back Projection*, *IR Iterative Reconstruction*

**Fig 4:**
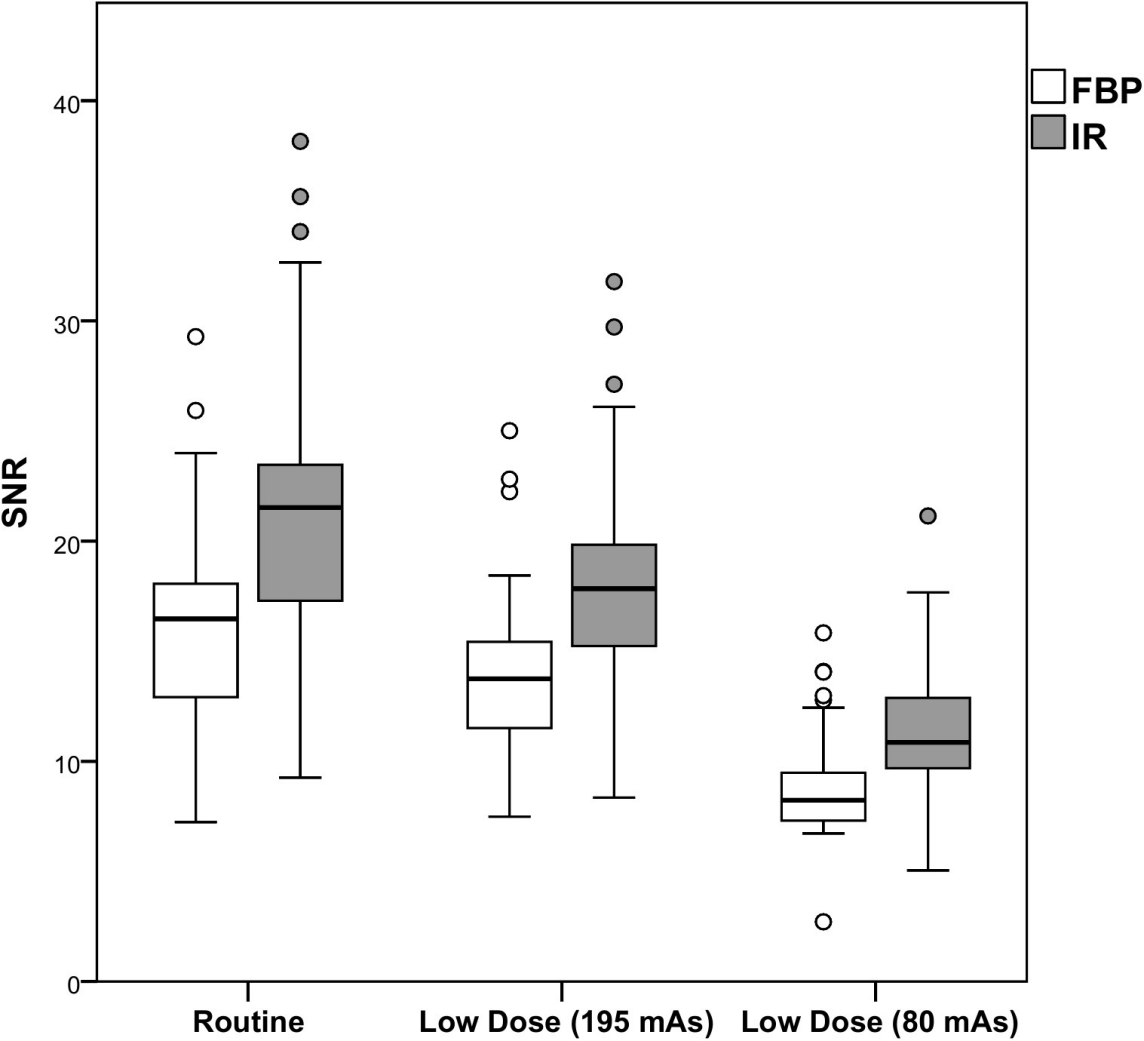
SNR per dose level for FBP and IR. The white boxes represent the SNR with FBP, while the gray boxes represent the SNR with IR. IR resulted in an increase in SNR compared to FBP at the same dose level. *FBP Filtered Back Projection*, *IR Iterative Reconstruction*, *SNR Signal-to-noise ratio*

**Fig 5:**
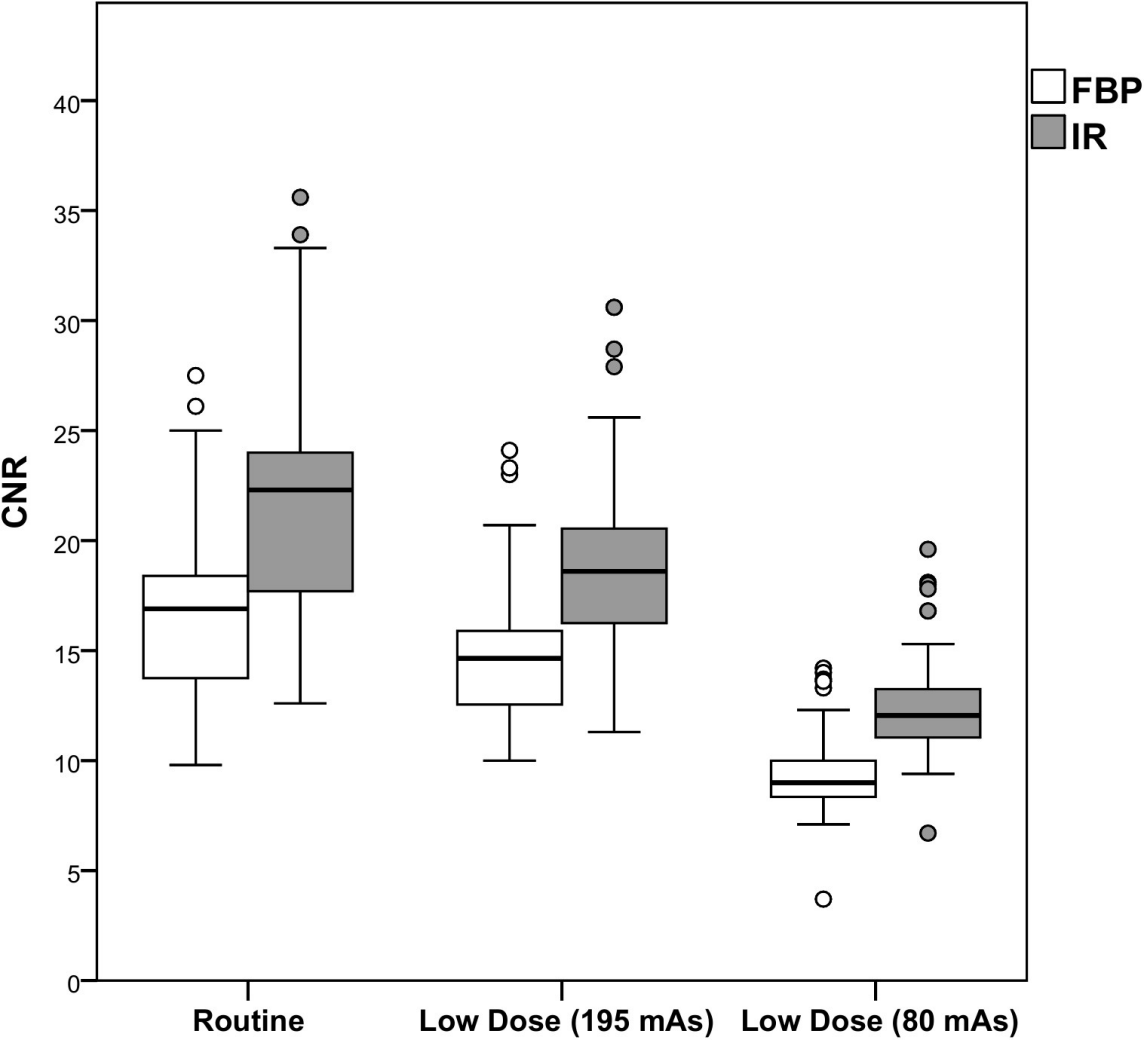
CNR per dose level for FBP and IR. The white boxes represent the CNR with FBP, while the gray boxes represent the CNR with IR. IR resulted in an increase in CNR compared to FBP at the same dose level. *CNR Contrast-to-noise ratio*, *FBP Filtered Back Projection*, *IR Iterative Reconstruction*

Results of the comparison with regard to objective image quality are shown in Figs 3–5 and Table 1. At each radiation dose level, IR resulted in improved objective image quality compared to FBP. Noise was lower at routine dose with IR (-21.6%, p<0.0005), but increased at both reduced dose levels with 49.6% and 143.9% (FBP) and with 17.5% and 86.6% (IR). The SNR and the CNR were improved both at routine dose and at 52% reduced dose with IR, while FBP and the lowest dose level resulted in a decrease in SNR and CNR (p<0.0005). Full data are provided in S1 Table.

**Table 1:** Objective image quality. Values are presented as median [interquartile range]. The relative difference is the percentage difference compared to FBP at routine dose (reference standard). The value represents the median relative change of the eight acquisitions.

	Dose	FBP	Relative change	IR	Relative change
**Noise**	Routine	34.9 [33.2–36.7]	NA	27.5 [26.0–28.8]*	-21.6%
	Low dose (80 kV, 195 mAs)	53.0 [48.0–55.5]*	49.6%	41.3 [37.4–43.1]*	17.5%
	Low dose (80 kV, 80 mAs)	87.7 [82.0–89.3]*	143.9%	66.9 [62.9–68.3]*	86.6%
**SNR**	Routine	16.48 [12.89–18.18]*	NA	21.52 [17.18–23.58]*	30.8%
	Low dose (80 kV, 195 mAs)	13.75 [11.48–15.46]*	-13.8%	17.84 [14.99–19.84]*	10.6%
	Low dose (80 kV, 80 mAs)	8.23 [7.30–9.53]*	-31.0%	10.86 [9.69–12.89]*	-46.5%
**CNR**	Routine	16.9 [13.7–18.4]*	NA	22.3 [17.7–24.0]*	29.3%
	Low dose (80 kV, 195 mAs)	14.7 [12.5–15.9]*	-12.1%	18.8 [16.4–20.6]*	11.9%
	Low dose (80 kV, 80 mAs)	9.0 [8.3–10.1]*	-44.6%	12.1 [11–13.4]*	-28.7%

NA not applicable

* p<0.01

### Subjective image quality

Inter-observer reliability for qualitative image quality scores was excellent (k = 0.81). The percentage inter-observer agreement was 93%. Subjective image quality scores are displayed in Table 2. Overall, the image quality was excellent (median 4.0) with no differences between the routine and low dose protocols or between FBP and IR (all p-values >0.05). Full data are provided in S2 Table.

**Table 2:** Subjective image quality scores. Scores are displayed as median [interquartile range]. No significant differences compared to FBP at routine dose were observed. *1 poor*, *non-diagnostic image quality*, *2 moderate*, *limited diagnostic value*, *3 good*, *diagnostic image quality*, *4 excellent*, *excellent image quality*

	FBP		IR	
	*Center*	*Outlets*	*Center*	*Outlets*
**Routine dose** **(100 kV, 195 mAs)**	4.0 [3.0–4.0]	4.0 [3.0–4.0]	4.0 [3.8–4.0]	4.0 [3.8–4.0]
**Low dose** **(80 kV, 195 mAs)**	4.0 [3.8–4.0]	4.0 [3.8–4.0]	4.0 [3.8–4.0]	4.0 [3.8–4.0]
**Low dose** **(80 kV, 80 mAs)**	4.0 [3.8–4.0]	4.0 [3.8–4.0]	4.0 [3.8–4.0]	4.0 [3.8–4.0]

## Discussion

This in-vitro study showed that for pediatric great vessel stent CT imaging a radiation dose reduction of more than 81% is feasible without affecting subjective image quality. Although the objective image quality decreased with both FBP and IR at this dose level, the subjective image quality remained excellent and the use of IR resulted in improved objective image quality compared with FBP.

The results of this study are relevant since risk estimates suggest that pediatric CT results in an increased radiation risk over adult CT [13]. This is worrisome, since the use of CT in pediatrics increased in the past decades [14]. To reduce the radiation dose burden, it is essential to increase awareness and decrease unnecessary CT examinations [15] as well as applying the As Low As Reasonably Achievable (ALARA) concept [16]. One of the strategies to achieve radiation dose reduction is applying IR techniques [8,17]. IR has also shown to allow for reduction of metal blooming artifacts, which is especially beneficial for the evaluation of stents [18]. Several studies have investigated the use of IR for stent evaluation, but mainly in coronary artery stents [19–24]. Ebersberger et al.[25] investigated 37 implanted coronary artery stents at full and half radiation dose and found improved objective image quality and comparable subjective image quality at reduced radiation dose using IR. However, a relatively high radiation dose was used of 4.3 mSv at half radiation dose. A study performed by Wuest and colleagues [22] in 73 implanted coronary stents at a radiation dose of 0.3 mSv (DLP 22.6 mGy*cm) found improved objective and subjective image quality with IR. Only one radiation dose level was used. To our best knowledge only one study investigated the effect of radiation dose reduction for great vessel stent imaging [26]. Two dose levels were used, namely 1.8 mSv (120 kVp, 80 mAs) and 0.6 mSv (80 kVp, 80 mAs) and results were compared to digital angiography. Both groups were comparable in body weight, age and stent size. There was good correlation with digital angiography at both dose levels. Subjective image quality was the same at the two dose levels, which is comparable to our study results.

In this study the dose was reduced with 52% and 81% compared to routine dose. However, routine dose levels may vary between hospitals. The current guidelines for aortic disease state that the estimated radiation dose of aortic CT is 10–15 mSv which is very high compared to current literature [27]. The American Association of Physicists in Medicine is currently working on reference protocols for CT, but there are no protocols for pediatric cardiac CT available yet. Our routine dose is however comparable to a previous study by Eichhorn et al. [26].

In our study the objective image quality decreased at low dose levels both with FBP and IR. A hybrid IR algorithm was used. Conventionally, FBP is used for image reconstruction, which creates images using projection data (backward projection). With true IR, both backward and forward projection steps are used, in which projection data are created using imaging data [28]. Hybrid iterative reconstruction is a blend of FBP and IR, and iterates in the projection data domain and image data domain. It reduces image noise because statistical properties of the acquisition are included in the reconstruction process. While model-based IR algorithms are more advanced and use both forward and backward projection steps between the projection domain and the image domain, thereby approaching true IR [6]. Further improvement in objective image quality can possibly be achieved with model-based IR algorithms which have shown to improve objective image quality further [6]. Another promising method to improve image quality further is the use of dual-energy CT which enables the acquisition of mono-energetic images at different keV levels. Using a high keV-level blooming artifacts can be almost completely suppressed, however at the cost of reduced stent visibility [20]. First results show improved stent lumen visualization in coronary stents, however this effect might be less pronounced for large vessels stents but is currently unknown [20].

To our best knowledge, this study is the first to systematically assess the potential of IR for great vessel stent imaging. Radiation dose can be drastically reduced without affecting subjective image quality, and IR can be used to improve objective image quality at low dose levels. Since aortic coarctation is a congenital disease, stents are mainly implanted at young age and multiple follow-up examinations are often required. Therefore, radiation dose reduction for this indication is very important for daily practice. The current study contributes to a further reduction in radiation dose of CT angiography examinations in children after stent implantation. This study has however several limitations. It concerns an *in vitro* study, therefore the effect of motion artifacts is unknown. However, because of the *in vitro* set up we were able to repeat acquisitions and investigate multiple dose levels using the same phantom. Because we were only interested in the image quality of the stent, a short scan length was used which is not representative for the clinical situation where a larger scan length is used to depict the surrounding anatomical structures. This will result in a lower effective dose than feasible in clinical practice, therefore the CTDIvol was presented. Furthermore, future research should determine the diagnostic accuracy for pathology, since in our study no abnormalities like in-stent stenosis were present. Finally, only one hybrid IR algorithm was used, and results may be different with other hybrid and model-based IR algorithms. However, the overall results could be generalizable to other vendors and algorithms as we found substantial dose reduction to be feasible with routine FBP using subjective image quality was used as endpoint.

In conclusion, this study showed that in an *in vitro* setting, substantial CT radiation dose reduction can be achieved for pediatric great vessel stent imaging without affecting the subjective image quality. Using IR techniques is helpful as a significant improvement of the objective image quality was achieved by noise reduction, which resulted in an increase of SNR and CNR. Future research should determine the diagnostic accuracy with reduced dose acquisitions in an in-patient setting.

## Supporting information

S1 Table Data objective image quality. (PDF)

S2 Table Data subjective image quality. (PDF)
