# Supplementary material for: Radiation dose reduction in pediatric great vessel stent computed tomography using iterative reconstruction: A phantom study
Source: PLoS One. 2017 Apr 14;12(4):e0175714. doi: 10.1371/journal.pone.0175714 (PMC5391930; doi:10.1371/journal.pone.0175714)
Supplement: S1 Table — (PDF) [file pone.0175714.s001.pdf]

Additional datafile. Values represent the objective image quality score for each dose level and reconstruction. Objective image quality was scored on all 8 acquisitions. *IR Iterative Reconstruction, FBP Filtered Back Projection*

Stent type	Acquisition	Routine dose 100 kV, 195 mAs FBP			Routine dose 100 kV, 195 mAs IR		
		Noise	SNR	CNR	Noise	SNR	CNR
Advanta V1	1	33,0	17,7	18,2	25,8	23,9	24,0
Advanta V1	2	33,7	18,3	18,4	26,5	19,8	21,3
Advanta V1	3	36,2	15,5	16,3	28,4	22,7	22,3
Advanta V1	4	33,8	17,0	17,6	26,5	22,1	22,7
Advanta V1	5	35,2	16,8	17,2	27,5	24,8	23,6
Advanta V1	6	34,5	18,8	18,4	26,9	22,1	22,6
Advanta V1	7	35,7	15,7	16,6	28,0	21,1	21,6
Advanta V1	8	38,1	16,3	16,4	29,9	20,1	20,6
AndraStent	1	38,6	13,9	13,8	30,4	17,9	17,6
AndraStent	2	37,3	13,8	13,8	29,3	17,8	17,8
AndraStent	3	37,6	13,0	13,3	29,5	18,5	18,1
AndraStent	4	37,7	14,1	13,9	29,5	18,6	18,1
AndraStent	5	36,6	14,2	14,2	28,7	17,5	17,8
AndraStent	6	38,1	13,8	13,9	29,9	17,1	17,3
AndraStent	7	36,4	12,9	13,5	28,6	16,6	17,3
AndraStent	8	37,9	15,9	14,9	29,7	21,2	19,3
Cheatham-	1	32,7	8,5	11,4	25,4	10,1	13,7
Cheatham-	2	34,8	7,2	9,8	27,7	10,5	14,0
Cheatham-	3	31,7	7,6	10,3	24,9	11,1	14,8
Cheatham-	4	29,5	10,4	13,7	23,1	11,0	14,7
Cheatham-	5	31,4	8,0	10,7	24,6	12,9	16,7
Cheatham-	6	32,0	9,9	12,9	25,1	14,0	18,1
Cheatham-	7	29,6	7,3	9,9	23,1	10,6	14,2
Cheatham-	8	28,9	8,2	11,1	22,6	9,3	12,6
IntraStent I	1	36,7	17,7	18,2	28,8	21,3	22,3
IntraStent I	2	36,9	17,2	17,7	28,9	22,9	23,0
IntraStent I	3	34,3	17,8	18,6	26,9	23,1	24,0
IntraStent I	4	36,2	18,7	18,6	28,4	21,8	22,7
IntraStent I	5	35,3	17,0	17,9	27,6	21,8	22,9
IntraStent I	6	35,7	16,7	17,6	28,1	24,7	24,3
IntraStent I	7	37,5	16,7	17,3	29,4	23,2	22,9
IntraStent I	8	35,8	17,1	17,8	28,0	21,7	22,8
Formula 53	1	33,0	23,3	24,7	25,8	34,0	33,9
Formula 53	2	33,3	24,0	25,0	26,0	23,3	26,9
Formula 53	3	35,0	21,8	23,2	27,5	31,8	31,8
Formula 53	4	33,2	25,9	26,1	26,0	23,7	27,5
Formula 53	5	33,6	22,6	24,2	26,4	38,2	35,6
Formula 54	6	33,9	29,3	27,5	26,7	32,7	32,7
Formula 54	7	34,0	11,2	14,2	26,9	35,6	33,3
Formula 54	8	34,5	21,4	22,9	27,1	28,4	29,8

Stent type	Acquisition	Low dose 80 kV, 195 mAs FBP			Low dose 80 kV, 195 mAs IR		
		Noise	SNR	CNR	Noise	SNR	CNR
Advanta V1	1	55,2	15,2	15,0	43,0	20,0	19,5
Advanta V1	2	54,7	15,5	15,3	42,7	15,5	17,0
Advanta V1	3	56,4	13,3	14,0	44,1	17,7	18,3
Advanta V1	4	53,3	13,3	14,2	41,6	19,8	19,7
Advanta V1	5	53,3	16,1	15,9	41,0	18,0	19,0
Advanta V1	6	49,7	13,8	15,0	38,7	18,4	19,7
Advanta V1	7	54,9	14,3	14,8	42,9	21,1	20,3
Advanta V1	8	53,1	13,7	14,5	41,4	17,2	18,4
AndraStent	1	56,8	12,3	12,4	45,5	16,7	16,1
AndraStent	2	58,8	10,4	11,2	44,2	13,5	14,6
AndraStent	3	58,6	11,5	11,7	44,4	16,2	16,0
AndraStent	4	56,4	12,3	12,5	50,3	15,7	14,9
AndraStent	5	60,5	11,6	11,7	44,4	15,0	15,5
AndraStent	6	57,3	12,2	12,2	45,8	16,1	15,9
AndraStent	7	63,5	11,9	11,6	44,4	17,8	16,9
AndraStent	8	57,3	13,5	13,0	missing	missing	missing
Cheatham-	1	48,9	8,9	11,6	38,2	11,6	15,1
Cheatham-	2	43,1	10,1	13,1	33,7	12,5	16,4
Cheatham-	3	48,1	9,1	11,9	37,4	13,3	17,0
Cheatham-	4	43,9	9,9	13,0	34,3	12,9	16,7
Cheatham-	5	45,7	9,7	12,6	36,1	13,4	17,1
Cheatham-	6	47,9	10,2	13,0	37,6	9,9	13,2
Cheatham-	7	45,6	7,5	10,0	36,0	13,0	16,7
Cheatham-	8	45,1	10,7	13,6	35,6	8,4	11,3
IntraStent I	1	49,6	14,8	16,1	38,7	19,6	21,1
IntraStent I	2	54,1	15,4	15,9	42,0	19,0	19,9
IntraStent I	3	52,8	14,6	15,5	41,3	19,0	20,1
IntraStent I	4	52,9	15,1	15,9	41,5	19,8	20,6
IntraStent I	5	53,7	15,1	15,8	42,0	16,7	18,4
IntraStent I	6	55,2	13,8	14,8	43,1	17,4	18,8
IntraStent I	7	53,0	13,1	14,5	41,3	19,6	20,5
IntraStent I	8	55,6	14,1	14,9	43,1	18,6	19,4
Formula 53	1	47,9	22,2	23,0	37,4	27,1	28,7
Formula 53	2	47,2	16,4	19,0	36,6	22,3	25,6
Formula 53	3	49,7	18,2	20,2	38,7	21,3	24,6
Formula 53	4	48,2	18,4	20,7	37,6	21,2	24,4
Formula 53	5	48,1	22,8	23,3	37,5	26,1	27,9
Formula 54	6	46,9	16,4	19,1	36,5	29,7	30,6
Formula 54	7	49,6	25,0	24,1	38,8	31,8	30,6
Formula 54	8	47,4	17,1	19,7	37,0	19,8	23,6

Stent type	Acquisition	Low dose 80 kV/ 80 mAs FBP			Low dose 80 kV/ 80 mAs IR		
		Noise	SNR	CNR	Noise	SNR	CNR
Advanta V1	1	88,1	8,4	9,0	67,0	10,9	11,7
Advanta V1	2	96,1	8,3	8,5	73,4	11,0	11,2
Advanta V1	3	88,5	9,1	9,1	67,7	12,0	12,1
Advanta V1	4	80,5	9,1	9,7	61,5	11,0	12,1
Advanta V1	5	97,3	8,4	8,5	74,6	13,2	12,1
Advanta V1	6	89,4	8,1	8,6	68,8	11,9	12,0
Advanta V1	7	87,8	9,0	9,2	67,1	9,9	11,0
Advanta V1	8	88,5	7,7	8,4	68,5	10,5	11,1
AndraStent	1	93,4	6,8	7,1	67,1	8,8	9,4
AndraStent	2	88,2	7,4	7,7	67,1	9,4	9,9
AndraStent	3	88,9	7,2	7,5	68,3	9,0	9,5
AndraStent	4	85,4	6,8	7,4	64,6	10,8	10,8
AndraStent	5	87,8	8,0	8,0	66,9	9,7	10,1
AndraStent	6	87,8	7,3	7,6	70,3	9,5	9,7
AndraStent	7	91,3	6,7	7,2	65,2	9,6	10,1
AndraStent	8	89,6	7,9	7,9	68,2	9,7	10,0
Cheatham-	1	91,1	8,3	9,6	70,1	9,8	11,7
Cheatham-	2	88,6	7,0	8,6	67,6	11,2	12,9
Cheatham-	3	85,1	7,5	9,0	64,9	5,0	6,7
Cheatham-	4	87,2	7,3	8,9	66,6	10,7	12,4
Cheatham-	5	84,7	8,2	9,6	64,8	10,0	12,1
Cheatham-	6	90,3	7,0	8,5	69,4	9,3	11,2
Cheatham-	7	86,2	6,7	8,3	65,9	9,1	11,3
Cheatham-	8	85,5	7,8	9,2	65,1	9,7	11,8
IntraStent I	1	78,8	9,4	10,2	59,8	12,9	13,9
IntraStent I	2	89,6	9,6	9,8	68,8	12,5	12,7
IntraStent I	3	84,1	7,3	8,5	64,2	13,1	13,5
IntraStent I	4	84,2	2,7	3,7	64,6	12,1	12,8
IntraStent I	5	87,8	8,8	9,3	67,1	10,7	11,8
IntraStent I	6	81,7	9,8	10,3	62,6	10,9	12,2
IntraStent I	7	87,5	8,2	9,0	67,0	10,6	11,6
IntraStent I	8	89,7	7,9	8,7	68,7	13,0	13,0
Formula 53	1	80,3	10,2	11,8	61,6	17,7	18,1
Formula 53	2	79,5	14,1	14,2	61,6	12,6	14,8
Formula 53	3	75,1	15,8	15,7	58,1	12,9	15,3
Formula 53	4	81,9	10,9	12,3	62,7	17,5	18,0
Formula 53	5	82,3	14,1	14,0	63,6	21,1	19,6
Formula 54	6	79,3	12,4	13,3	61,0	15,2	16,8
Formula 54	7	78,8	12,8	13,7	60,5	17,0	17,8
Formula 54	8	78,7	13,0	13,6	60,3	15,2	16,8
