# Supplementary material for: Radiation dose reduction in pediatric great vessel stent computed tomography using iterative reconstruction: A phantom study
Source: PLoS One. 2017 Apr 14;12(4):e0175714. doi: 10.1371/journal.pone.0175714 (PMC5391930; doi:10.1371/journal.pone.0175714)
Supplement: S2 Table — (PDF) [file pone.0175714.s002.pdf]

Additional datafile. Values represent the subjective image quality score (two observers) for each dose level and reconstruction. *IR Iterative Reconstruction, FBP Filtered Back Projection*

Stent type	Rater (1 or 2)	Routine dose 100 kV, 195 mAs FBP Center	Routine dose 100 kV, 195 mAs FBP Outlets	Routine dose 100 kV, 195 mAs IR Center	Routine dose 100 kV, 195 mAs IR Outlets
Advanta V12 covered	1	4	4	4	4
AndraStent 30-XL	1	4	4	4	4
Cheatham-Platinum	1	2	3	3	3
IntraStent Max LD	1	4	4	4	4
Formula 535	1	4	4	4	4
Advanta V12 covered	2	4	4	4	4
AndraStent 30-XL	2	3	3	4	4
Cheatham-Platinum	2	3	3	3	3
IntraStent Max LD	2	4	4	4	4
Formula 535	2	4	4	4	4

Stent type	Rater (1 or 2)	Low dose 80 kV, 195 mAs FBP Center	Low dose 80 kV, 195 mAs FBP Outlets	Low dose 80 kV, 195 mAs IR Center	Low dose 80 kV, 195 mAs IR Outlets
Advanta V12 covered	1	4	4	4	4
AndraStent 30-XL	1	4	4	4	4
Cheatham-Platinum	1	2	3	3	3
IntraStent Max LD	1	4	4	4	4
Formula 535	1	4	4	4	4
Advanta V12 covered	2	4	4	4	4
AndraStent 30-XL	2	4	4	4	4
Cheatham-Platinum	2	3	3	3	3
IntraStent Max LD	2	4	4	4	4
Formula 535	2	4	4	4	4

Stent type	Rater (1 or 2)	Low dose 80 kV/ 80 mAs FBP Center	Low dose 80 kV/ 80 mAs FBP Outlets	Low dose 80 kV/ 80 mAs IR Center	Low dose 80 kV/ 80 mAs IR Outlets
Advanta V12 covered	1	4	4	4	4
AndraStent 30-XL	1	4	4	4	4
Cheatham-Platinum	1	3	3	3	3
IntraStent Max LD	1	4	4	4	4
Formula 535	1	4	4	4	4
Advanta V12 covered	2	4	4	4	4
AndraStent 30-XL	2	4	4	4	4
Cheatham-Platinum	2	3	3	3	3
IntraStent Max LD	2	4	4	4	4
Formula 535	2	4	4	4	4
